# Comparison of cytokine levels in the aqueous humor of polypoidal choroidal vasculopathy and neovascular age-related macular degeneration patients

**DOI:** 10.1186/s12886-019-1278-8

**Published:** 2020-01-08

**Authors:** Huiying Zhou, Xinyu Zhao, Mingzhen Yuan, Youxin Chen

**Affiliations:** Department of Ophthalmology, Peking Union Medical College Hospital, Peking Union Medical College, Chinese Academy of Medical Sciences, No.1 Shuaifuyuan, Wangfujing, Dongcheng District, 100730 Beijing, China

**Keywords:** Aqueous humor, Cytokines, Age-related macular degeneration, Polypoidal choroidal vasculopathy

## Abstract

**Background:**

The concentrations of cytokines in the aqueous humor from neovascular age-related macular degeneration (nAMD) and polypoidal choroidal vasculopathy (PCV) may vary. The study was conducted to compare various cytokine levels in the aqueous humor of eyes with PCV, nAMD and control.

**Methods:**

The present case control study included 49 treatment-naïve eyes from 49 patients (PCV 24, nAMD 11, and cataract 14 eyes). Totally 34 angiogenic and inflammatory cytokines in the aqueous humor were measured by Luminex bead-based multiplex array.

**Results:**

After adjusting for gender and age by multivariate logistic analysis, concentrations of IL-31, LIF, SDF1-α, VEGF-A, VEGF-D were significantly higher in eyes with nAMD or PCV compared with control eyes (all *P* < 0.05, times in nAMD: 59.5, 6.0, 7.0, 4.5, 5.6, respectively, times in PCV: 51.9, 5.21, 6.6, 4.0, 5.1, respectively), and concentrations of HGF, IP-10, MCP-1, IL-13 were significantly lower in eyes with nAMD or PCV than in control eyes (all *P* < 0.05, times in nAMD: 2.6, 2.0, 4.5, 4.7, respectively, times in PCV: 1.9, 3.0, 3.0, 2.8, respectively), but none of the 34 cytokines, including VEGF and IL-8, showed significantly different between eyes with nAMD and PCV.

**Conclusions:**

Various cytokines involved in inflammation and angiogenesis including elevated IL-31, LIF, SDF1-α, VEGF-A, VEGF-D might be involved in the pathogenesis of nAMD or PCV. None of the 34 cytokines may help to differentiate nAMD and PCV.

## Background

Age-related macular degeneration (AMD) is a leading cause of irreversible visual disability and blindness among elderly in developed countries. Clinically, AMD is divided into two major types, the non-neovascular AMD and neovascular AMD (nAMD), and the latter is the major cause of severe visual loss. Polypoidal choroidal vasculopathy (PCV) is characterized by branching choroidal networks with polyp-like aneurysmal dilation, which can be clearly demonstrated by indocyanine green angiography (ICGA). PCV shares many similarities with nAMD, including clinical manifestations, genetic background, and both of them are choroidal vasculopathy associated with subretinal hemorrhage, scars and fibrosis [1], so some views support PCV is a subtype of nAMD. However, they still differ in pathophysiology, histopathological, epidemiology, some clinical characteristics, treatment responses, natural course and special genes [2–6]. There remain controversies as to whether PCV is merely a variant of nAMD or its own distinct entity [7]. Since PCV is mostly common in Asians, recent information supports the notion that angiogenesis and inflammation play an important and perhaps central role in the pathogenesis of nAMD and PCV [8]. There are some literatures on cytokines profile in aqueous humor of nAMD and PCV [9–11], but relatively few studies investigated the differences of both angiogenic and inflammatory cytokines in the aqueous humor among nAMD, PCV and controls. In the past, with the limitation of traditional ELISA consumables and aqueous humor contents, detecting the concentrations of cytokines in aqueous humor were restricted on a few selected cytokines [12, 13]. While the use of Luminex assay, a commercially available multiplex immunoassay with the advantages of high sensitivity and high throughput, makes evaluating a panel of cytokines in a small number of clinical samples comes to truth. Therefore, in this study, we first aim to further discover the differences between nAMD with PCV by analyzing cytokine profile. Next, we explored other new potential therapeutic targets besides blocking VEGF therapy via detecting various angiogenic and inflammatory cytokines levels in aqueous humor of nAMD and PCV.

## Methods

This study was reviewed and approved by the Ethics Committee in Peking Union Medical College Hospital and adhered to the tenets of the Declaration of Helsinki. Written informed consent was obtained from all participants.

### Patients

Forty-nine patients including 24 with treatment-naive PCV, 11 patients with treatment-naive nAMD and 14 controls without any other associated sight-threatening pathology except for cataract who had been scheduled to undergo cataract surgery were recruited from the Department of Ophthalmology, Peking Union Medical College Hospital. All patients with PCV and nAMD were diagnosed specifically with fluorescein angiography (FFA) and ICGA in active stage with distinct lesions which were scheduled to accept anti-VEGF intravitreal injection. Eyes with PCV were indicated with clusters of polypoidal dilation of the vessels with or without abnormal vascular networks in the superficial choroid. Eyes with nAMD showed classic choroidal neovascularization (CNV) or occult CNV with FFA, with no polypoidal lesions in ICGA. Controls were selected among the patients undergoing cataract surgery. All collected subjects met inclusion criteria, which had no history or slit-lamp evidence of ocular trauma, no use of systemic corticosteroids, immunosuppressants, or antimetabolites, and no unrelated ocular diseases like any types of retinal diseases, pathological myopia, glaucoma, uveitis, vitreous hemorrhage, choroiditis, hereditary diseases and previous intraocular surgery.

### Sample extraction and preparation

The aqueous humor samples were collected at the beginning of cataract surgery in the controls and at the time of anti-VEGF intravitreal injection into the eyes with PCV or nAMD. After topical anesthesia, approximately 100 μl aqueous humor was withdrawn aseptically using an insulin syringe with a 30-gauge needle at the corneal limbus and was rapidly frozen and stored at − 80 °C until final measurement.

### Measurement of cytokines

The concentrations of the cytokines in the aqueous humor samples were measured with Luminex platform following the manufacturer’s instructions. We measured 34 cytokines associated with inflammation and angiogenesis, including interleukin (IL)-1α, IL-1β, IL-1RA, IL-2, IL-4, IL-5, IL-6, IL-7, IL-8, IL-9, IL-10, IL-13, IL-15, IL-17A, IL-18, IL-21, IL-22, IL-23, IL-27, IL-31, VEGF-A, VEGF-D, leukemia inhibitory factor (LIF), stromal-derived factor 1-α (SDF1-α), brain-derived neurotrophic factor (BDNF), interferon inducible protein 10(IP-10), hepatocyte Growth Factor (HGF), interleukin-12p70(IL-12p70), PIGF, basic fibroblast growth factor (FGF-basic), granulocyte macrophage colony stimulating Factor (GMCSF), epidermal growth factor (EGF), macrophage inflammatory protein 1β(MIP-1β), Eotaxin, regulated upon the activation of normal T cell expressed and secreted (RANTES), platelet-derived growth factor-BB (PDGF-BB), MCP-1, nerve growth factor b(NGFb), stem cell factor (SCF), macrophage inflammatory protein-1α(MIP-1α), growth-related gene product α (GROα), interferon-γ (IFNγ), interferon-α(IFNα), tumor necrosis factor (TNF)α and TNFβ. Numbers on the Luminex plates were read by using Magpix system (Luminex Corp., Austin, TX, USA) following the manufacturer’s instructions, and Bio-Plex manager 6.1 software (Bio-Rad Laboratories, Hercules, CA, USA) with a five-parameter curve-fitting algorithm was used to analyze the data.

### Statistical analysis

Statistical analyses were performed using SPSS 16.0 software for the calculation of the mean and SD of the cytokines. Differences between each pair of groups including PCV, neovascular AMD and controls were assessed by the ANOVA test or Mann-Whitney U test for continuous variables and by the Chi-square test for categorical variables. ANOVA test used for data measurement when data meet normal distribution, Mann-Whitney test used when data were abnormal distribution. Multivariate logistic regression analysis was performed to confirm the association of cytokines with nAMD or PCV and look for the differences between nAMD and PCV after adjusted age and gender. Bonferroni correction used when gender was abnormal distribution. *P* < 0.05 was deemed to be statistically significant except when using Mann-Whitney test which *P* < 0.0167 was deemed to be statistically significant.

## Results

The details for all subjects including sex and age were listed in Table 1. The average age of the patients with nAMD, PCV and control was 75.55 ± 6.83 (mean ± SD), 67.46 ± 8.79, 70.07 ± 12.65 years old, respectively. The mean age had no statistical difference among the 3 groups. Men in AMD, PCV and control group were 5 (45.6%), 21 (87.5%) and 4 (28.6%), respectively. However, gender showed statistical difference among 3 groups(*P* < 0.05). Table 2 shows the concentrations of the 34 cytokines in the aqueous humor samples. Among the 34 cytokines, the aqueous humor levels of IL-8 in the nAMD group were significantly higher than the PCV group (*p* = 0.012). The levels of IL-18, IL-21, IL-31, LIF, SDF1-α, FGF-basic, VEGF-A, and VEGF-D in the aqueous humor were significantly higher in the nAMD group than the control (p = < 0.0001, < 0.0001, < 0.0001, < 0.0001, < 0.0001, < 0.0001, < 0.0001, < 0.0001, respectively). Concentrations of IL-8, IL-18, IL-21, IL-31, LIF, SDF1-α, FGF-basic, VEGF-A, and VEGF-D in the PCV group were significantly higher than the control (*p* = 0.012, < 0.0001, < 0.0001, < 0.0001, < 0.0001, < 0.0001, 0.001, < 0.0001, < 0.0001, respectively). In contrast, the levels of BDNF, HGF, IP-10, MCP-1, IL-13 and IL-17A in the aqueous humor were significantly lower in the nAMD group than control (p = < 0.0001, < 0.0001, < 0.0001, < 0.0001, < 0.0001, 0.008, respectively). In PCV group, the levels of BDNF, GMCSF, HGF, IP-10, MCP-1, IL-12p70, IL-13 and IL-17A were significantly lower than control (*p* = < 0.0001, < 0.0001, < 0.0001, < 0.0001, < 0.0001, < 0.0001, < 0.0001, 0.008, respectively). After adjusting for gender and age by multivariate logistic analysis (Table 3), concentrations of IL-31, LIF, SDF1-α, VEGF-A, VEGF-D were significantly higher in eyes with nAMD or PCV compared with control eyes (all *P* < 0.05, times in nAMD: 59.5, 6.0, 7.0, 4.5, 5.6, respectively, times in PCV: 51.9, 5.21, 6.6, 4.0, 5.1, respectively), and concentrations of HGF, IP-10, MCP-1, IL-13 were significantly lower in eyes with nAMD or PCV than in control eyes (all P < 0.05, times in nAMD: 2.6, 2.0, 4.5, 4.7, respectively, times in PCV: 1.9, 3.0, 3.0, 2.8, respectively), but none of the 34 cytokines, including VEGF and IL-8, showed significantly different between eyes with nAMD and PCV.

**Table 1:** Demographic characteristics of patients

	nAMD (*n* = 11)	PCV (*n* = 24)	Controls (*n* = 14)
Male gender	5	21	4
P* value versus controls	0.434	< 0.001	/
P* value versus PCV	0.015	/	/
Mean age ± SD, years	75.55 ± 6.83	67.46 ± 8.79	70.07 ± 12.65
*p* value versus controls	0.988	0.940	/
p value versus PCV	0.986	/	/

*After Bonferroni correction

SD = standard deviation

**Table 2:** Levels of 34 cytokines (means ± SD) in the aqueous humor among 3 groups

cytokines	nAMD (pg/ml)	PCV (pg/ml)	Control (pg/ml)	P value (nAMD vs Control)	P value (PCV vs Control)	P value (nAMD vs PCV)
BDNF	4.58 ± 2.90	4.82 ± 2.83	18.69 ± 4.58	**< 0.0001**	**< 0.0001**	0.846
EGF	4.34 ± 2.65	4.07 ± 3.29	5.36 ± 2.72	0.403	0.208	0.807
Eotaxin	20.09 ± 3.96	17.64 ± 5.64	18.42 ± 6.03	0.449	0.671	0.221
GMCSF	31.62 ± 18.47	20.04 ± 17.93	40.07 ± 11.66	0.211	**0.001**	0.061
HGF	238.12 ± 113.63	325.59 ± 191.52	624.95 ± 165.93	**< 0.0001**	**< 0.0001**	0.165
IL-1RA	99.54 ± 31.25	89.35 ± 38.98	96.04 ± 37.40	0.815	0.593	0.453
IP-10	49.57 ± 36.70	33.44 ± 29.05	100.99 ± 38.55	**< 0.0001**	**< 0.0001**	0.195
MCP-1	61.57 ± 43.04	91.56 ± 88.82	274.24 ± 63.01	**< 0.0001**	**< 0.0001**	0.271
MIP-1α	8.91 ± 4.55	8.94 ± 5.46	8.72 ± 5.48	0.930	0.900	0.985
MIP-1β	50.91 ± 10.24	53.34 ± 17.27	55.47 ± 12.22	0.443	0.667	0.650
IL-2	0.32 ± 0.24	0.31 ± 0.24	0.32 ± 0.25	0.993	0.948	0.943
IL-4	0.03 ± 0.01	0.03 ± 0.01	0.03 ± 0.01	0.755	0.766	0.944
IL-5	0.07 ± 0.06	0.09 ± 0.05	0.08 ± 0.05	0.559	0.386	0.151
IL-6	0.72 ± 0.55	0.79 ± 0.48	0.96 ± 0.53	0.259	0.327	0.728
IL-8*	40.68 ± 35.47	14.63 ± 29.94	0.58 ± 0.60	0.053	**0.012**	**0.012**
IL-12p70*	2.89 ± 1.99	1.17 ± 1.67	3.49 ± 0.34	0.236	**< 0.0001**	0.029
IL-13	1.36 ± 1.26	2.25 ± 1.62	6.40 ± 1.58	**< 0.0001**	**< 0.0001**	0.120
IL-15	0.02 ± 0.01	0.02 ± 0.01	0.02 ± 0.01	0.553	0.835	0.642
IL-17A*	1.05 ± 0.60	1.30 ± 0.84	2.27 ± 1.65	**0.008**	**0.008**	0.370
IL-18*	40.98 ± 32.69	51.18 ± 37.16	0.41 ± 0.32	**< 0.0001**	**< 0.0001**	0.938
IL-21*	22.30 ± 20.41	14.78 ± 16.20	0.00 ± 0.00	**< 0.0001**	**< 0.0001**	0.243
IL-23	0.05 ± 0.02	0.03 ± 0.02	0.04 ± 0.03	0.622	0.182	0.078
IL-27	0.04 ± 0.03	0.05 ± 0.03	0.05 ± 0.03	0.802	0.980	0.764
IL-31*	10.11 ± 6.34	8.83 ± 5.13	0.17 ± 0.49	**< 0.0001**	**< 0.0001**	0.559
LIF*	87.17 ± 8.54	74.76 ± 36.89	14.33 ± 5.15	**< 0.0001**	**< 0.0001**	0.508
NGFβ	11.10 ± 6.64	9.78 ± 6.55	8.82 ± 5.09	0.365	0.646	0.562
PDGF-BB	1.90 ± 0.77	1.68 ± 0.88	1.62 ± 0.90	0.426	0.845	0.482
PIGF	0.28 ± 0.20	0.25 ± 0.14	0.26 ± 0.14	0.806	0.852	0.657
RANTES	4.49 ± 3.74	6.65 ± 4.26	4.23 ± 3.17	0.873	0.070	0.132
SCF*	5.75 ± 3.34	23.66 ± 3.34	5.89 ± 3.92	0.947	0.041	0.185
SDF1-α*	285.56 ± 73.64	273.10 ± 135.54	41.10 ± 10.58	**< 0.0001**	**< 0.0001**	0.755
FGF-basic*	0.11 ± 0.14	0.05 ± 0.10	0.00 ± 0.00	**< 0.0001**	**0.001**	0.031
VEGF-A*	175.71 ± 36.29	154.43 ± 87.56	38.69 ± 17.26	**< 0.0001**	**< 0.0001**	0.483
VEGF-D*	242.49 ± 28.96	221.50 ± 96.24	43.66 ± 23.61	**< 0.0001**	**< 0.0001**	0.459

SD = Standard deviation. IL = Interleukin, LIF = leukemia inhibitory factor, SDF1-α = stromal-derived factor 1-α, BDNF = brain-derived neurotrophic factor, IP-10 = interferon inducible protein 10, HGF = hepatocyte Growth Factor, IL-12p70 = interleukin-12p70, PIGF = placental growth factor, FGF-basic = basic fibroblast growth factor, GMCSF = granulocyte macrophage colony stimulating Factor, EGF = epidermal growth factor, MIP-1β = macrophage inflammatory protein 1β, RANTES = regulated upon the activation of normal T cell expressed and secreted, PDGF-BB = platelet-derived growth factor-BB, MCP-1 = monocyte chemoattractant protein, NGFb = nerve growth factor b, SCF = stem cell factor, MIP-1α = macrophage inflammatory protein-1α, GROα = growth-related gene product α, IFNγ = interferon-γ, IFNα = interferon-α, TNF = tumor necrosis factor. Bold = P value was statistically significant(P < 0.05 was deemed to be statistically significant which using ANOVA test when data meet normal distribution, while * means unnormal distribution including IL-8, IL-12p70, IL-17A, IL-18, IL-21, IL-31, LIF, SCF, SDF1-α, FGF-basic, VEGF-A and VEGF-D using Mann-Whitney test which P < 0.0167 was deemed to be statistically significant)

**Table 3:** Association of nAMD or PCV with aqueous humor cytokines (multivariate logistic analysis)

	HGF	IP-10	MCP-1	IL-13	IL-31	LIF	SDF1-α	VEGF-A	VEGF-D
nAMD (vs. control)									
P value	0.002	0.007	0.004	0.017	0.014	0.003	0.021	0.001	0.002
OR	0.987	0.957	0.959	0.026	8.902	1.099	1.037	1.045	1.034
	(0.978–0.995)	(0.927–0.988)	(0.932–0.987)	(0.001–0.516)	(1.552–51.065)	(1.033–1.170)	(1.005–1.069)	(1.018–1.073)	(1.012–1.056)
PCV (vs. control)									
P value	0.006	0.001	0.012	0.032	0.017	0.007	0.021	0.003	0.004
OR	0.99	0.946	0.975	0.041	8.411	1.076	1.037	1.039	1.028
	(0.983–0.997)	(0.915–0.979)	(0.956–0.994)	(0.002–0.763)	(1.468–48.198)	(1.020–1.135)	(1.005–1.069)	(1.013–1.066)	(1.009–1.047)

*p* values are adjusted for age and gender. OR = Odds ratio. Figures in parentheses indicate 95% confidence intervals

## Discussion

In the present study, we investigated the levels of 34 cytokines in aqueous humor from treatment-naïve eyes with nAMD, PCV and controls, and explored whether there were differences among the three groups. Our results revealed that there were nine cytokines levels associated with nAMD and PCV. Among them, IL-31, LIF, SDF1-α, VEGF-A, VEGF-D levels were significantly higher in eyes with nAMD or PCV compared with control eyes, while HGF, IP-10, MCP-1, IL-13 were significantly lower in nAMD and PCV in contrast with controls. In present study, nAMD group showed no statistically significant difference with PCV group after adjusted age and gender, which was consistent with previous studies, although the IL-8 concentration was significantly higher in nAMD than in PCV [13–15]. Agrawal et al. [14] investigated 41 different cytokines from 16 nAMD patients, 18 PCV patients and 50 age- and sex-matched cataract patients supported that there were no significant differences in cytokine levels observed between nAMD and PCV patients for aqueous humor, while MIP-1α were significantly higher in AMD patients than controls and MIP-1α, IL-8, IP-10 and MCP levels were significantly higher in PCV patients than controls. Moreover, Sakurada et al. [13] studied 14 cytokines, including VEGF, none of them were significantly different between eyes with nAMD and those with PCV, although they reported biomarkers of inflammation, elevated CRP and IP-10, and suggested chronic inflammation might be involved in the pathogenesis of nAMD or PCV. The differences among those studies maybe limited sample sizes and variety of cytokines. IL-8 are the chemokines produced by macrophages and other cells, which are primarily involved in the regulation of inflammatory responses, moreover, previous study revealed that elevated intraocular levels of IL-8 and IL-8 gene polymorphisms were associated with angiogenesis [16]. Balne et al. and Mimura et al. [10, 15] reported that aqueous humor cytokines IL-8 levels was significantly higher in nAMD and PCV patients than controls which was consistent with our finding although the difference was not obvious after multivariate logistic analysis. It may be the potential cytokine to distinguish from normal and explain the differences of nAMD and PCV, so further studies are necessary to assess whether it plays a pathogenetic role in nAMD and PCV.

We found that some cytokines levels in the aqueous humor of nAMD and PCV patients had seldom been reported in the past. Some pro-inflammatory and pro-angiogenic cytokines were increased in nAMD and PCV groups compared with controls, such as SDF1-α, IL-31, LIF, VEGF-D. SDF1-α, a potent stimulator of VEGF expression, acting as an angiogenic agent increased during inflammation, is the main candidate factor of neovascularization and vascular permeability [17, 18]. IL-31, an immunoregulatory protein belonging to the pro-inflammatory IL-6 cytokine family [19], might inhibit angiogenesis [19]. LIF, a member of the IL-6 family of cytokines, upregulated with inflammation having the function of both anti-angiogenic and pro-angiogenic, was reported to have the ability to protect the integrity of the vasculature and retinal away from degenerations [20, 21]. The function of these cytokines on the progress of the eyes of nAMD and PCV need further research in the future, which may offer alternative therapeutic approaches to treat visual defects associated with nAMD and PCV. VEGF-D and VEGF-A had been identified to play an important role in angiogenesis. The previous study reported that the high expression of VEGF-D, in addition to VEGF-A, exacerbated the angiogenic response of retinal pigment epithelium (RPE) cell in nAMD [22]. However, as far as we know, there was few research on the concentration of VEGF-D in PCV, let alone the comparison of aqueous humor levels of VEGF-D in three groups, even they seldomly distinguished VEGF family such as detecting VEGF-A and VEGF-D respectively [13, 15]. Our study reported that the mean aqueous humor concentration of not only VEGF-A but also VEGF-D tended to be higher in both nAMD and PCV than that in controls. According to these findings, we had reasons to suspect the possibility that VEGF-D expression in eye besides VEGF-A modify the ocular angiogenesis as angiogenic stimulators in both nAMD and PCV. Therefore, agent combined with anti-VEGFs such as OPT-302 (to inhibit VEGF-C and VEGF-D) may be one of the emerging anti-VEGF treatments in the future [23]. Nowadays, we should pay more attention to the function and concentration of VEGF-D in aqueous humor of nAMD and PCV. Thus, we may infer that development of procedures to neutralize elevated cytokines in the eye with nAMD or PCV may potentially be therapeutic targets, as anti-VEGF drugs to neutralize VEGF.

Some pro-inflammatory and pro-angiogenic cytokines had no change in nAMD and PCV groups compared with controls like IL-6. IL-6 may cause a breakdown of the blood-ocular barrier by inducing an increase of endothelial permeability, and promote CNV. While our study showed no differences between nAMD, PCV and control group, which is in agreement with prior research [24]. However, IL-6 was significantly higher in the AMD group than in the cataract group [10].

Some pro-inflammatory and pro-angiogenic cytokines were dramatically decreased in nAMD and PCV groups compared with controls, such as MCP-1, HGF, IL-13, IP-10. MCP-1, a potent factor that regulated the migration and infiltration of monocytes and macrophages, enhanced by pro-inflammatory molecules, and facilitated angiogenesis [25]. Previous studies have reported inconsistent results on the level of MCP-1. Some suggested that MCP-1 levels in the aqueous humor in eyes with nAMD or PCV were not significantly different from controls [10, 26, 27]. However, some investigators observed that MCP-1 levels in the aqueous humor showed statistically significant elevation in the AMD group compared with control group and then put forward that MCP-1 may be one of the future targets for the treatment of AMD [28–30]. Therefore, more research is still needed for the function and concentration of MCP-1. HGF was identified to stimulate the proliferation, migration, neovascularization and differentiation, which played an important role in ocular angiogenesis and increases leakage from retinal vessels [31]. Dramatically, in our study, aqueous humor concentration of HGF did show a significantly lower trend in nAMD and PCV patients compared with controls. IL-13 played pathophysiological roles in allergic inflammation and fibrosis formation. In the present study, IL-13 was significantly lower in the nAMD and PCV group than in the cataract group, while reports of aqueous humor IL-13 level in nAMD or PCV are still controversial according to recent reports. Fu and colleagues found that IL-13 of aqueous humor was significantly upregulated during AMD development, suggesting that IL-13 could have potent effects on the pathological process of this disease [12]. However, according to another research, aqueous humor levels of IL-13 showed no significant difference between AMD group and the cataract group [10], while there are few reports about IL-13 level in aqueous humor of PCV patient. Therefore, whether IL-13 makes sense in the pathogenic processes of nAMD and PCV or not still needs to be clarified in further research. IP-10 concentrations were reduced in the aqueous humor in eyes with nAMD and PCV in contrast to control group in the current study. However, some previous reports demonstrated that IP-10 expression was increased in AMD and PCV patients, highlighting the involvement of inflammatory pathways [13, 15, 24, 26, 32]. On the other hand, Funk et al. [33] reported that IP-10 concentrations in aqueous humor with AMD were similar to those in controls. This indicates that further studies are necessary to illuminate the role of IP-10 in the pathogenic process of nAMD and PCV.

Limitations of our study should be mentioned. Firstly, the main limitation of this study was the small number of subjects, which meant that our findings should be confirmed in a larger number of patients. Secondly, gender bias wasn’t taken into account when compared differences between groups because of disability to match gender and expand sample size. Moreover, another limitation was that we did not compare the relationships between structure, function and aqueous humor cytokines concentrations, future experiments should be conducted on animals and cells level to assess the effects of differential molecules. Besides, we didn’t take into account of the exact typing of the classic AMD or atypical AMD. Furthermore, as we reported that inflammation and angiogenesis may have a common influence on the pathogenesis of nAMD and PCV, a treatment response targeting angiogenesis and inflammation should raise concern in the future study. Last but not least, there are maybe some other cytokines related to nAMD and PCV that was not covered in the present study, such that exploring new and more related-cytokines is needed in the future.

## Conclusion

Our research studied the concentrations of 34 cytokines in aqueous humor of patients with nAMD, PCV and the controls. Findings of our study which have not been widely reported in previous researches were elevated intraocular levels of IL-31, LIF, SDF-α and VEGF-D, which may be associated with the pathogenesis of nAMD and PCV. Importantly, these various pro-inflammatory and pro-angiogenic cytokines may be potential novel therapeutic target for nAMD and PCV. Moreover, we found significant difference in aqueous humor IL-8 levels between nAMD and PCV although the difference was not obvious after adjusted age and gender, and these inflammatory factors deserved further concern in future larger sample sizes and age- and gender-matched researches.

## Data Availability

The datasets used and analyzed during the current study are available from the corresponding author upon reasonable request.

## Abbreviations

AMD: Age-related macular degeneration
BDNF: brain-derived neurotrophic factor
CNV: choroidal neovascularization
EGF: epidermal growth factor
FFA: fluorescein angiography
FGF-basic: basic fibroblast growth factor
GMCSF: granulocyte macrophage colony stimulating Factor
GROα: growth-related gene product α
HGF: hepatocyte Growth Factor
ICGA: indocyanine green angiography
IFNα: interferon-α
IFNγ: interferon-γ
IL: Interleukin
IL-12p70: interleukin-12p70
IP-10: interferon inducible protein 10
LIF: leukemia inhibitory factor
MCP-1: monocyte chemoattractant protein
MIP-1α: macrophage inflammatory protein-1α
MIP-1β: macrophage inflammatory protein 1β
nAMD: neovascular age-related macular degeneration
NGFb: nerve growth factor b
PCV: polypoidal choroidal vasculopathy
PDGF-BB: platelet-derived growth factor-BB
PIGF: placental growth factor
RANTES: regulated upon the activation of normal T cell expressed and secreted
SCF: stem cell factor
SDF1-α: stromal-derived factor 1-α
TNF: tumor necrosis factor
